# Highly sensitive electrochemical determination of cariprazine using a novel Ti_3_C_2_@CoAl_2_O_4_ nanocomposite: application to pharmaceutical and biological sample analysis

**DOI:** 10.1007/s00604-025-07104-1

**Published:** 2025-03-24

**Authors:** Elif Naz Öven, Asena Ayse Genc, Nevin Erk, Wiem Bouali, Qamar Salamat, Mustafa Soylak

**Affiliations:** 1 Department of Analytical Chemistry, Faculty of Pharmacy, Ankara University, 06560 Ankara, Turkey; 2 The Graduate School of the Health Sciences, Ankara University, 06110 Ankara, Turkey; 3 Department of Chemistry, Faculty of Sciences, Erciyes University, 38039 Kayseri, Turkey; 4 Technology Research & Application Center (TAUM), Erciyes University, 38039 Kayseri, Turkey; 5 Turkish Academy of Sciences (TUBA), Cankaya, Ankara, Turkey

**Keywords:** Cariprazine, Electrochemistry, Nanocomposite, Modified electrode, Differential pulse voltammetry, Rare disease

## Abstract

Cariprazine (CAR) is an atypical antipsychotic drug used for the treatment of schizophrenia and bipolar disorder. This study presents the development of a novel, highly sensitive electrochemical sensor based on a Ti_3_C_2_@CoAl_2_O_2_ nanocomposite–modified glassy carbon electrode (GCE) for the detection of CAR in pharmaceutical and biological samples. The innovative Ti_3_C_2_@CoAl_2_O_2_ composite, synthesized and characterized through Fourier-transform infrared spectroscopy, X-ray diffraction, scanning electron microscopy coupled with energy-dispersive spectroscopy, and thermogravimetric analysis, revealed exceptional structural integrity, morphology, composition, and thermal stability. The electrochemical properties of the modified electrode were evaluated using cyclic voltammetry and electrochemical impedance spectroscopy, demonstrating enhanced conductivity, an increased electroactive surface area, and reduced charge transfer resistance compared to the bare GCE. Differential pulse voltammetry was employed for CAR detection under optimized conditions, yielding a linear range of 0.2–5.6 μM with a regression equation *I*_pa_ (μA) = 0.133 *C*_CAR_ (μM) + 0.09 (*R*^2^ = 0.993). The limit of detection and limit of quantification were determined as 0.02 µM and 0.07 µM, respectively, highlighting the sensor’s high sensitivity. The modified electrode exhibited excellent repeatability with a relative standard deviation (RSD) of = 2.9% and reproducibility (RSD = 2.8%), along with strong selectivity against common interfering substances. The sensor was successfully applied to human blood serum, urine, and CAR tablets, achieving high recovery values (98.52–103.94%), confirming its reliability for real-sample analysis. These findings underline the novelty and potential of the Ti_3_C_2_@CoAl_2_O_2_-modified GCE as a powerful tool for the accurate, selective, and sensitive determination of CAR in clinical and pharmaceutical applications.

**Graphical Abstract:**

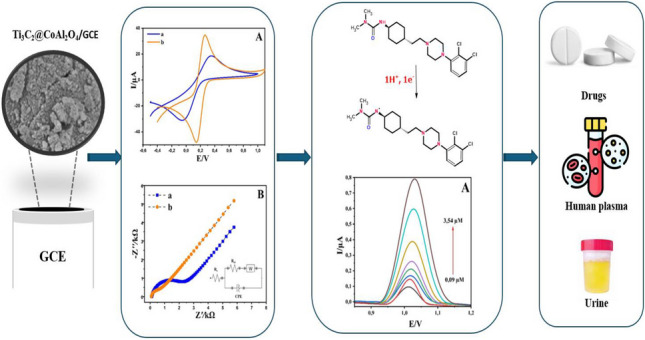

**Supplementary Information:**

The online version contains supplementary material available at 10.1007/s00604-025-07104-1.

## Introduction

Schizophrenia is a severe psychiatric disorder that affects both cognitive and emotional functions, often accompanied by symptoms such as hallucinations, delusions, thought disorders, social withdrawal, and emotional blunting [1]. Comorbid conditions like substance use disorders, mood disorders, anxiety disorders, and obsessive–compulsive disorders frequently complicate its management [2].

Cariprazine (CAR), an atypical antipsychotic, has demonstrated efficacy in treating schizophrenia and bipolar disorder by balancing dopamine (D2/D3 receptors) and serotonin (5-HT1A receptors) activity in the brain. The typical maintenance dose ranges from 3 to 6 mg per day, depending on patient response and tolerance [3].

The pharmacokinetics of oral CAR can be characterized using a three-compartment model, which assumes that the drug undergoes first-order processes for both absorption and elimination. In contrast, the pharmacokinetics of its primary active metabolites, desmethyl cariprazine and didesmethyl cariprazine, are better described by a two-compartment model with linear elimination [4].

It is important to note that it takes approximately five half-lives for CAR to reach steady-state concentrations. As a result, the drug level increases gradually over several weeks, even if the daily dose remains unchanged. Clinicians who are unfamiliar with this process may prematurely increase the dose before reaching steady state, potentially overshooting the optimal dose and requiring later dose reduction. Furthermore, when CAR is discontinued, the active drug remains in the system for several weeks before complete elimination. This prolonged elimination may be beneficial in managing schizophrenia, a condition often associated with poor medication adherence. Missing an occasional dose may have a lower risk of relapse compared to drugs with shorter half-lives, although this potential benefit has not yet been confirmed in clinical trials [5].

The efficacy and safety of CAR have been so far investigated only in a few short-term (unpublished) clinical trials; however, three studies in schizophrenia and three studies in bipolar mania/mixed episodes evidenced a statistically significant therapeutic effect compared to placebo for CAR at doses ranging from 1.5 to 12 mg day^−1^. There does not appear to be clinically relevant adverse effects of CAR on metabolic variables [6].

Traditional analytical methods for the detection and quantification of CAR, such as high-performance liquid chromatography [7], spectrofluorimetry [8], liquid chromatography–tandem mass spectrometry [9], and high-performance thin-layer chromatography [10], are well-established techniques that provide high precision and reliability. However, these methods are often resource intensive, requiring specialized equipment, trained personnel, and significant analysis time. Moreover, they can be costly and are typically not feasible for use in routine analysis or in laboratories with limited resources.

In contrast, electrochemical methods present a compelling alternative due to their numerous advantages. These techniques are cost-effective, relatively simple to implement, and provide rapid analytical results [11, 12]. They do not require the extensive sample preparation or complex instrumentation typically associated with traditional methods, making them more accessible and suitable for a broader range of applications, particularly in settings with limited funding or resources [13, 14].

Among the various electrochemical techniques, differential pulse voltammetry (DPV) stands out as one of the most widely used methods for electrochemical sensing due to its high sensitivity and precision. Due to its exceptional sensitivity, the DP technique is particularly advantageous for trace-level analysis, making it highly suitable for applications in pharmaceutical, forensic, and environmental studies. Given its affordability and accessibility, DPV is widely employed for detecting drug active compounds in pharmaceutical formulations and biological fluids. One of its most significant benefits is its ability to achieve remarkably low detection limits, enhancing the accuracy and reliability of analytical measurements across various applications [15–17].

Moreover, cyclic voltammetry (CV) is widely recognized as a fundamental technique for the preliminary electrochemical investigation of new materials and systems. It plays a crucial role in analyzing complex electrode reactions by providing valuable insights into their mechanisms. In particular, CV profiles offer essential information regarding electron transfer kinetics, thermodynamic parameters, and the overall behavior of the electrochemical process, making it an indispensable tool in sensor development and material characterization [18–20].

Recent advancements in electrochemical sensor technology have focused on integrating nanomaterials and polymers to improve sensitivity, selectivity, and detection limits. The electrode, a critical component of electrochemical sensors, plays a pivotal role in determining performance. Key factors include electrode type, properties of modified materials, and their interaction with the electrode surface [21–23].

Glassy carbon electrode (GCE) is one of the most widely utilized electrodes in electrochemical drug detection due to its exceptional properties. Its broad potential window, high electrical conductivity, reusability, and remarkable mechanical and chemical stability make it a preferred choice for analytical applications, particularly in the development of sensitive and reliable electrochemical sensors [24, 25].

Nanoscale metals and their oxides (e.g., Cu, Fe, Co, Mn, Ni) exhibit diverse applications, attributed to their small particle size, enhanced surface properties, stability, and excellent thermal and electrical conductivity. Among these, spinel structures (AB₂O₄) are particularly notable for their superior electrocatalytic performance, thermal stability, and mechanical robustness. In this category, cobalt aluminate (CoAl₂O₄), often referred to as blue spinel, stands out due to its high oxygen ion mobility, large surface area, favorable electrochemical characteristics, and environmentally friendly nature [26]. The incorporation of Co^2^⁺ and Al^3^⁺ ions not only enhances electrical conductivity but also increases the electroactive surface area and the density of active sites, thereby improving electrocatalytic efficiency [27].

To further enhance electrochemical properties, the integration of highly conductive materials such as Ti₃C₂ MXene with transition metal aluminates creates a synergistic effect, leading to improved electron transfer kinetics, lower charge transfer resistance, and higher sensitivity. MXene materials (Mn + 1AXn), derived from selective etching of A-layer elements, exhibit exceptional properties due to their unique layered structure and coexistence of metallic, ionic, and covalent bonds. Titanium carbide (Ti_3_C_2_), a widely studied MXene, offers excellent electrical conductivity, a large specific surface area, and robust physical and chemical stability, making it a promising candidate for sensor applications [28].

The objective of this study is to develop a novel electrochemical sensor based on Ti₃C₂@CoAl₂O₄/GCE for the sensitive detection and quantification of CAR. The Ti₃C₂@CoAl₂O₄ nanocomposite was characterized through X-ray diffraction (XRD), scanning electron microscopy–energy-dispersive spectrometry (SEM–EDS), thermogravimetric analysis (TGA), CV, and electrochemical impedance spectroscopy (EIS) to assess its structural, morphological, compositional, and electrochemical properties. This work not only introduces a new composite material but also demonstrates its superior electrochemical performance as a sensor, highlighting its remarkable sensitivity, selectivity, and exceptionally low detection limits for CAR in pharmaceutical and biological samples.

## Experimental

### Apparatus and chemicals

Information on the apparatus and chemicals used is provided in the Supporting Information.

### *Synthesis of Ti*_*3*_*C*_*2*_*@CoAl*_*2*_*O*_*4*_* nanocomposite*

#### ***Synthesis of cobalt (II) aluminate (CoAl***_***2***_***O***_***4***_***) spinel nanocrystals***

The synthesis of cobalt (II) aluminate (CoAl_2_O_4_) was carried out according to the previous study [29], with some modifications made as follows: First, the precursor solutions were prepared. To achieve this, 1.74 g of cobalt nitrate hexahydrate (Co (NO_3_)_2_·6H_2_O) and 4.5 g of aluminum nitrate nonahydrate (Al (NO_3_)_3_·9H_2_O) are combined with 4.53 g of oxalic acid (H_2_C_2_O_4_·2H_2_O). Oxalic acid plays a critical role in this synthesis as a complexing agent. It reacts with the metal ions to form soluble metal–oxalate complexes, which ensure that the Co^2+^ and Al^3+^ ions are evenly distributed throughout the mixture. This prevents local inhomogeneities and unwanted premature precipitation of metal hydroxides, ensuring better control over the stoichiometry and uniformity of the final product. Additionally, oxalic acid aids in facilitating the thermal decomposition of the precursor material during calcination, as it breaks down into gases (CO_2_ and CO) and leaves behind the desired oxide phases.

After adding the reagents, 40 mL of water is introduced to dissolve the chemicals and create a homogenous solution. The mixture is stirred thoroughly to ensure that all the components are completely dissolved, forming a clear and uniform precursor solution. At this stage, the pH of the solution is gradually raised by adding 10–12 mL of ammonia solution (NH_3_). This step is critical as it raises the pH to approximately 12, creating an alkaline environment that induces the precipitation of metal hydroxides. The hydroxides are formed to act as intermediates in the synthesis process, which will later convert to oxides during calcination. Stirring is continued for an additional 5 min to ensure that the reaction is complete and the precipitate is uniformly formed.

Once the precipitation step is completed, the product is washed sequentially with water and ethanol. After washing, the product is dried in an oven to remove any remaining solvents, leaving behind a dry precursor material. The final step in the synthesis is calcination, which is carried out by placing the dried precursor material in a furnace set to 700 °C for 180 min. Calcination serves multiple purposes. First, it facilitates the thermal decomposition of the metal hydroxides and any oxalate species present, transforming them into their respective oxides. During this process, CoO and Al_2_O_3_ are formed, which then react through solid-state diffusion to produce the CoAl_2_O_4_ spinel phase. The high temperature not only drives the formation of the spinel structure but also promotes the growth and crystallization of the nanocrystals, improving their phase purity and structural integrity. The duration and temperature of calcination are carefully chosen to optimize the crystalline quality while maintaining the nanoscale size of the particles.

#### ***Synthesis of titanium carbide (Ti***_***3***_***C***_***2***_***) MXene***

The synthesis of titanium carbide (Ti_3_C_2_) MXene [30] begins with the selective etching of aluminum (Al) atoms from the MAX phase precursor, Ti_3_AlC_2_. To initiate the synthesis, 1.0 g of Ti_3_AlC_2_ is combined with 4.14 g of lithium fluoride (LiF). LiF serves as the fluoride source, essential for producing hydrofluoric acid (HF) in situ when it reacts with hydrochloric acid (HCl). This reaction facilitates the removal of the Al layers through a controlled chemical process. The mixture is stirred for 5 min to ensure proper dispersion of the LiF and MAX phase particles, allowing optimal interaction during the subsequent reaction. Next, 40 mL of concentrated HCl, typically 35–37%, is added to the mixture. Upon addition, the HCl reacts with LiF to generate HF in situ. The HF formed plays a critical role in the selective etching process. It reacts with the Al atoms in the Ti_3_AlC_2_ MAX phase, breaking the strong chemical bonds between the Al layers and the Ti_3_C_2_ carbide layers.

The Al is removed as soluble aluminum fluoride (AlF_3_), leaving behind a layered Ti_3_C_2_ structure with expanded interlayer spacing. This reaction is conducted at an elevated temperature of 50 °C under continuous stirring for 48 h. The elevated temperature and prolonged reaction time are critical for ensuring complete etching of the Al layers and uniform MXene formation. After the etching process, the resulting Ti_3_C_2_ material is washed several times with water to remove residual salts, unreacted HCl, and any byproducts, such as AlF_3_ and lithium chloride (LiCl). The washing process is repeated until the pH of the supernatant reaches neutral (approximately pH 6–7), indicating that the excess acidic components have been thoroughly removed. This step is crucial to ensure the stability and purity of the synthesized MXene. Finally, the obtained material was placed in an oven to be dried at 90 °C for 20 h. At the end of the washing process, the Ti_3_C_2_ MXene typically exists as a slurry of nanosheets. These nanosheets possess a characteristic 2D structure with high surface area, hydrophilicity, and functional surface groups (e.g., –F, –OH, and –O) introduced during the etching process. These surface functional groups are important as they influence the material's properties, such as electrical conductivity, ion intercalation, and chemical stability, making Ti_3_C_2_ MXene highly versatile for a wide range of applications.

#### ***The synthesis of Ti***_***3***_***C***_***2***_***@CoAl***_***2***_***O***_***4***_*** nanocomposite***

To synthesize the Ti_3_C_2_@CoAl_2_O_4_ nanocomposite, 0.75 g of CoAl_2_O_4_ spinel nanocrystals and 0.75 g of Ti_3_C_2_ MXene were dispersed in 60 mL of a mixed solvent comprising ethanol and deionized water in a 1:1 ratio. The mixture was subjected to sonication for 25 min to achieve uniform dispersion of the components within the solvent. Following sonication, the solution was magnetically stirred at room temperature (RT) for 24 h to enhance homogeneity. Upon completion of the stirring process, the nanocomposite was separated via centrifugation. The resulting Ti_3_C_2_@ CoAl_2_O_4_ nanocomposite was then dried in an oven at 80 °C for 12 h to eliminate any residual solvent.

### *Fabrication Ti*_*3*_*C*_*2*_*@CoAl*_*2*_*O*_*4*_* nanocomposite*

The GCE surface was initially polished using an alumina slurry, followed by thorough rinsing with a 1:1 (v/v) mixture of deionized water and ethanol to remove any residual alumina particles. A suspension of Ti_3_C_2_@CoAl_2_O_4_ (1 mg mL^−1^) was prepared in a water–ethanol mixture, and 2 μL of this suspension was carefully drop-cast onto the cleaned GCE surface. The modified electrode was then subjected to infrared (IR) radiation for 10 min to facilitate drying. This procedure resulted in the successful fabrication of the Ti_3_C_2_@ CoAl_2_O_4_/GCE, which was subsequently used for the electrochemical detection of CAR.

### Preparation of real samples for analysis

The Ti_3_C_2_@ CoAl_2_O_4_ modified GCE developed in this study was successfully utilized for the detection of CAR in pharmaceutical preparations, human plasma, and human urine samples. Spiked sample preparation was carried out according to the protocol outlined in our previous study [31].

## Results and discussion

### *Characterization of Ti*_*3*_*C*_*2*_*@CoAl*_*2*_*O*_*4*_* nanocomposite*

The provided FTIR, XRD, SEM, EDS [32], and TGA spectra collectively illustrate the structural, chemical, morphological, compositional, and thermal characteristics of CoAl_2_O_4_, Ti_3_C_2_, and the Ti_3_C_2_@CoAl_2_O_4_ nanocomposite. These analyses provide comprehensive insight into the successful formation of the composite, the retention of structural and functional integrity in its components, and its overall stability and properties.

**Fig. 1:**
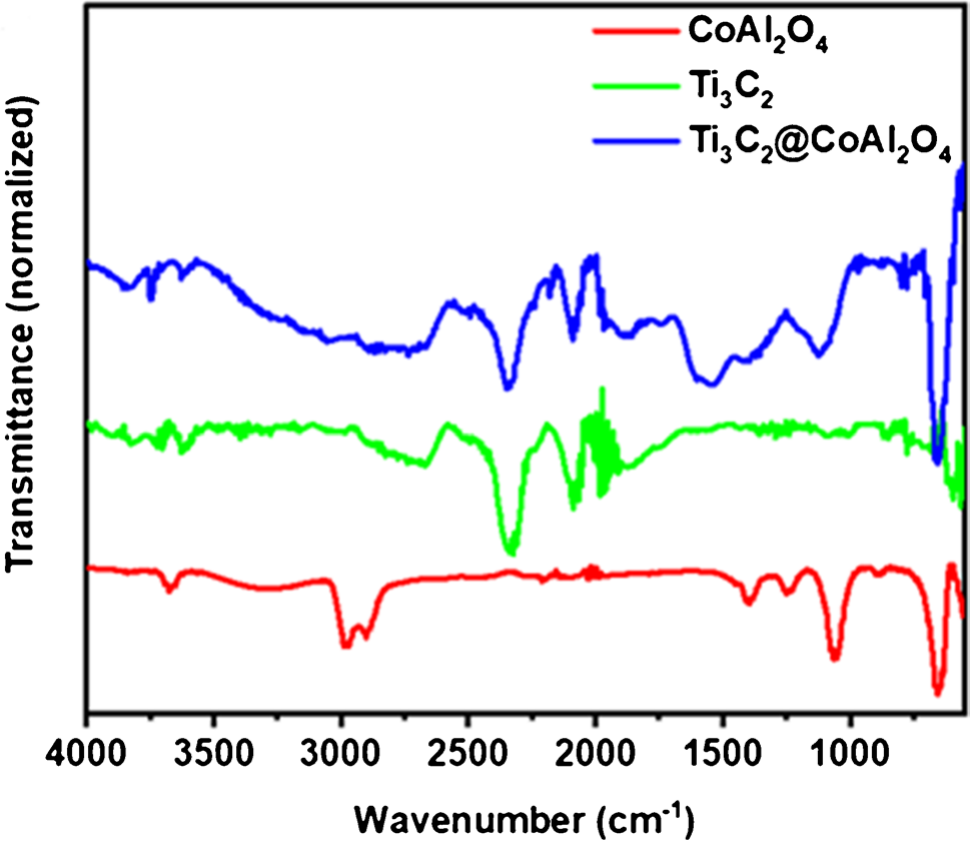
FTIR spectra of prepared CoAl_2_O_4_, Ti_3_C_2_ MXene, and Ti_3_C_2_@CoAl_2_O_4_ nanocomposite

The FTIR spectrum provides information about the functional groups present in the samples (Fig. 1). For CoAl_2_O_4_, the red spectrum shows distinct peaks below 1000 cm^−1^, which correspond to the Co–O and Al–O stretching vibrations. These peaks are characteristic of the spinel structure and confirm the presence of the metal–oxide bonds within CoAl_2_O_4_. Additionally, the absence of significant peaks in the higher wavenumber region suggests minimal organic or hydroxyl content in the spinel nanocrystals [33]. In contrast, the green spectrum for Ti_3_C_2_ MXene reveals peaks in the region of 3300–3600 cm^−1^, attributed to the stretching vibrations of hydroxyl groups (–OH) present on the surface of the MXene layers. This observation is consistent with the common surface functional groups (–OH, –F, and –O) introduced during the synthesis of Ti_3_C_2_ through chemical etching. Another significant peak near 1600 cm^−1^ is associated with C = O or Ti–O bonds, further confirming the presence of these surface terminations [34]. The blue spectrum of the Ti_3_C_2_@ CoAl_2_O_4_ composite incorporates features of both CoAl_2_O_4_ and Ti_3_C_2_. The Co–O and Al–O stretching vibrations below 1000 cm^−1^ are preserved, signifying that the spinel structure remains intact within the composite. Simultaneously, the –OH stretching vibrations near 3300 cm^−1^ from Ti_3_C_2_ are also present, indicating the retention of functional groups from the MXene component. The absence of any new peaks or significant shifts suggests that no major chemical changes occur during the formation of the composite, and the two materials are successfully integrated without altering their fundamental chemical structures.

**Fig. 2:**
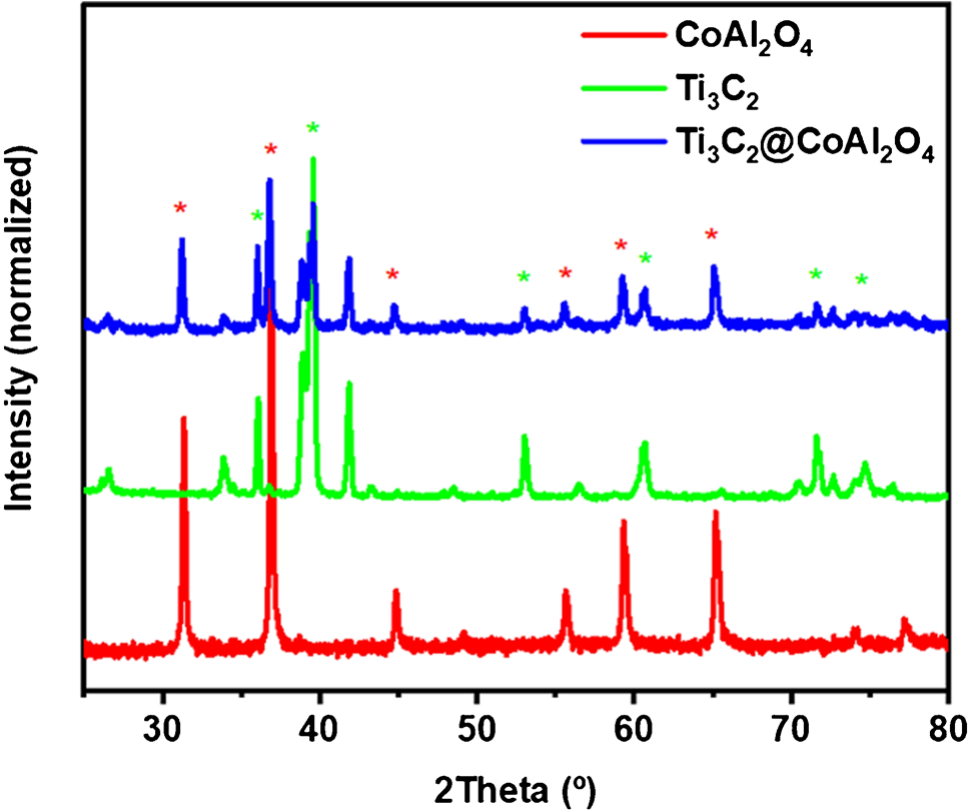
XRD patterns of synthesized CoAl_2_O_4_, Ti_3_C_2_ MXene, and Ti_3_C_2_@ CoAl_2_O_4_ nanocomposite

The XRD patterns provide a detailed analysis of the crystallographic structure of CoAl_2_O_4_, Ti_3_C_2_, and their composite (Fig. 2). In the red spectrum for CoAl_2_O_4_, sharp and well-defined peaks are observed at 2θ positions around 31°, 36°, 44°, and 59°, which are characteristic of the cubic spinel structure. The presence of these peaks indicates that CoAl_2_O_4_ exhibits high crystallinity, an essential property for maintaining its structural integrity in the composite. The green spectrum for Ti_3_C_2_ MXene highlights its characteristic layered structure [35]. The most notable features are the peaks, which appear around 37, 40, 53, 61, 72, and 76° that correspond to the hexagonal crystal structure of Ti_3_C_2_. The absence of peaks associated with the MAX phase precursor, Ti_3_AlC_2_, confirms that aluminum was completely removed during the synthesis process [36]. The blue spectrum for the Ti_3_C_2_@CoAl_2_O_4_ composite shows the retention of characteristic peaks from both CoAl_2_O_4_ and Ti_3_C_2_, confirming that the composite contains both components in their original crystalline forms. The lack of new diffraction peaks implies that no additional crystalline phases are formed during the composite synthesis. This demonstrates that the integration process did not alter the crystallographic structures of CoAl_2_O_4_ or Ti_3_C_2_, further validating the successful fabrication of the composite. To further analyze the crystallinity of the synthesized materials, the crystallite sizes of CoAl_2_O_4_, Ti_3_C_2_, and Ti_3_C_2_@CoAl_2_O_4_ were estimated using Scherrer’s equation. The calculated sizes were 10.51, 50.16, and 14.33 nm, respectively. The slightly larger crystallite size of the composite compared to pure CoAl_2_O_4_ can be attributed to interfacial interactions between Ti_3_C_2_ and CoAl_2_O_4_, which may lead to partial agglomeration or enhanced structural ordering. Despite this increase, the composite retains a significantly smaller crystallite size than bulk Ti_3_C_2_, which contributes to its improved electrochemical performance by increasing the effective surface area and enhancing charge transfer kinetics.

**Fig. 3:**
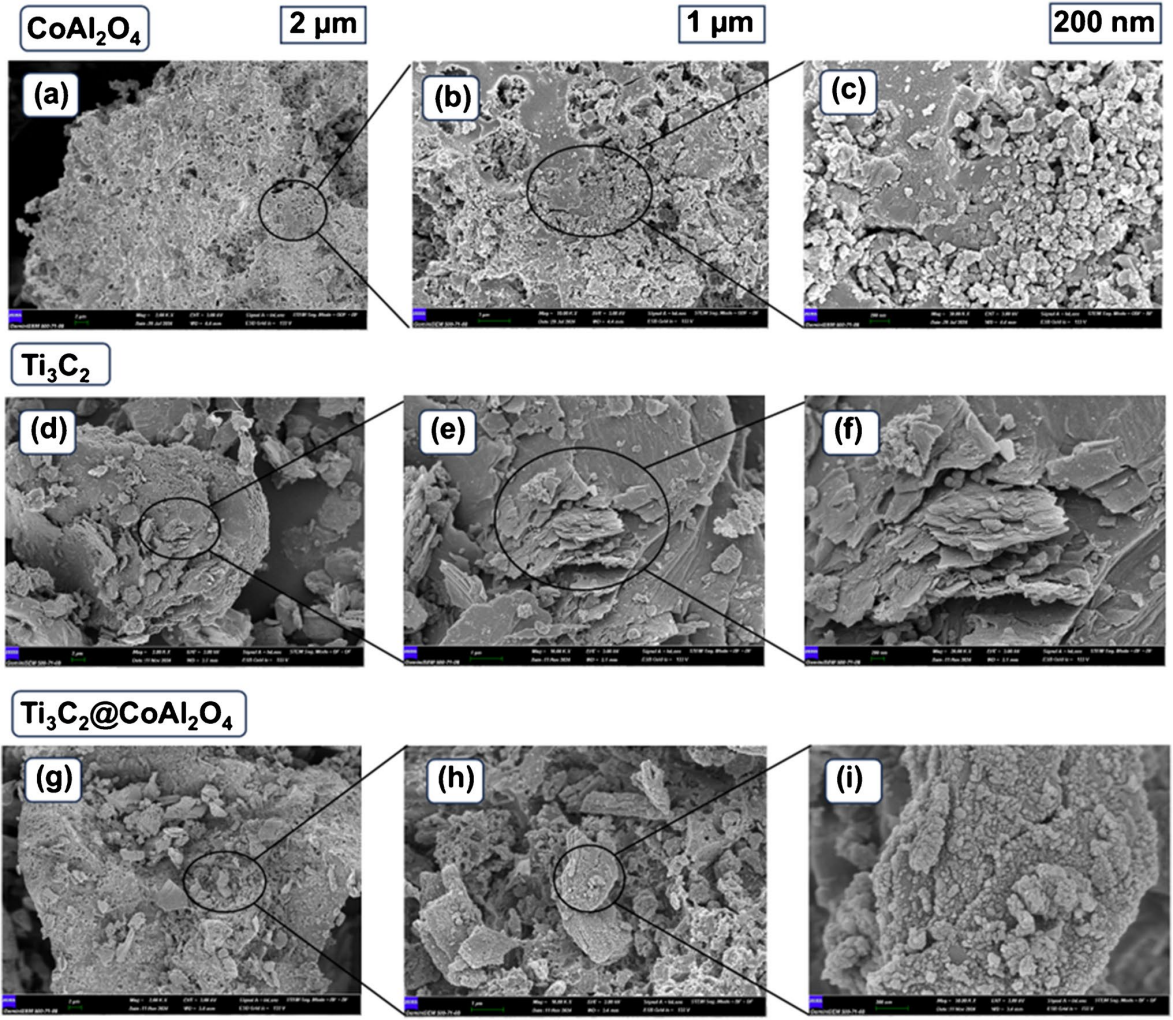
SEM images of as-prepared CoAl_2_O_4_ (a–c) [37], as-prepared Ti_3_C_2_ MXene (**d**–**f**), and Ti_3_C_2_@CoAl_2_O_4_ nanocomposite (**g**–**i**)

The SEM images of CoAl_2_O_4_ show aggregated irregularly shaped particles. At higher magnifications, the surface appears rough and granular, indicative of its spinel nanocrystal morphology. The individual particles are small, which is consistent with the high surface area characteristic of CoAl_2_O_4_ nanocrystals. This morphology plays a critical role in enhancing the composite’s interaction with other components, such as Ti_3_C_2_ (Fig. 3).

**Fig. 4:**
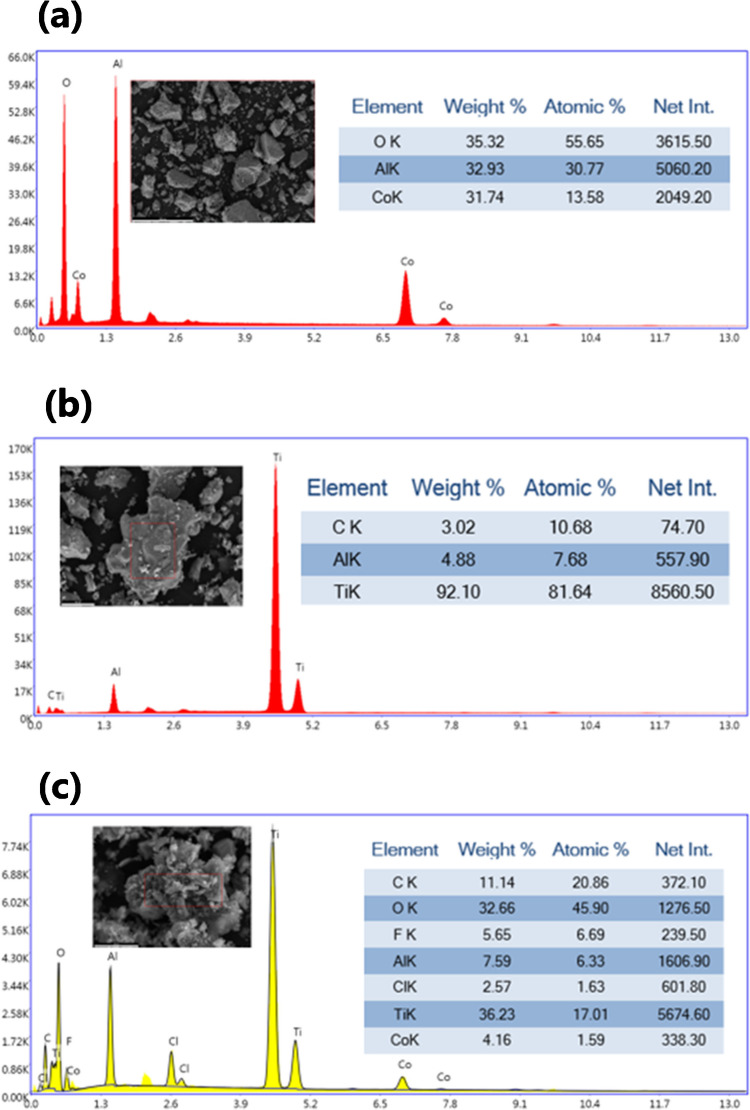
SEM–EDS analysis and the elemental composition of (**a**) CoAl_2_O_4_, (**b**) Ti_3_C_2_ MXene, and (**c**) Ti_3_C_2_@CoAl_2_O_4_ nanocomposite

Regarding the SEM images of Ti_3_C_2_ MXene, they exhibit the typical layered structure, which is a hallmark of MXenes. The exfoliated sheets are visible, with a stacked or accordion-like arrangement. This morphology provides a high specific surface area and excellent accessibility for interaction with other materials. The layered nature also contributes to the excellent conductivity and mechanical flexibility of Ti_3_C_2_ (Fig. 4a). Considering the Ti_3_C_2_@CoAl_2_O_4_ nanocomposite, SEM images reveal a well-integrated structure where CoAl_2_O_4_ nanoparticles are uniformly distributed over the surface of Ti_3_C_2_ nanosheets. The granular morphology of CoAl_2_O_4_ is retained, while the layered structure of Ti_3_C_2_ remains intact, indicating minimal disruption during the composite synthesis. This uniform distribution ensures strong interfacial contact between the two materials, which is essential for the enhanced properties of the composite (Fig. 4b).

The EDS spectrum for CoAl_2_O_4_ confirms the presence of cobalt (Co), aluminum (Al), and oxygen (O) as the main elements. Their atomic percentages align with the stoichiometry of CoAl_2_O_4_. The absence of significant impurities highlights the purity of the synthesized spinel nanocrystals. The spectrum for Ti_3_C_2_ identifies titanium (Ti), carbon (C), and oxygen (O) as the primary elements. The carbon content originates from the Ti_3_C_2_ structure, while the oxygen is attributed to the surface functional groups (e.g., –O, –OH, and –F) introduced during the etching process. The high titanium content further confirms the successful synthesis and preservation of the MXene structure. The EDS spectrum of the composite confirms the presence of titanium (Ti), cobalt (Co), aluminum (Al), carbon (C), and oxygen (O). The detection of all these elements indicates the successful integration of CoAl_2_O_4_ and Ti_3_C_2_ in the composite. The elemental ratios are consistent with the expected proportions of the two components, confirming the uniform distribution observed in the SEM images. Additionally, the trace amounts of fluorine (F) likely originate from the Ti_3_C_2_ synthesis process involving LiF and HCl (Fig. 4c).

**Fig. 5:**
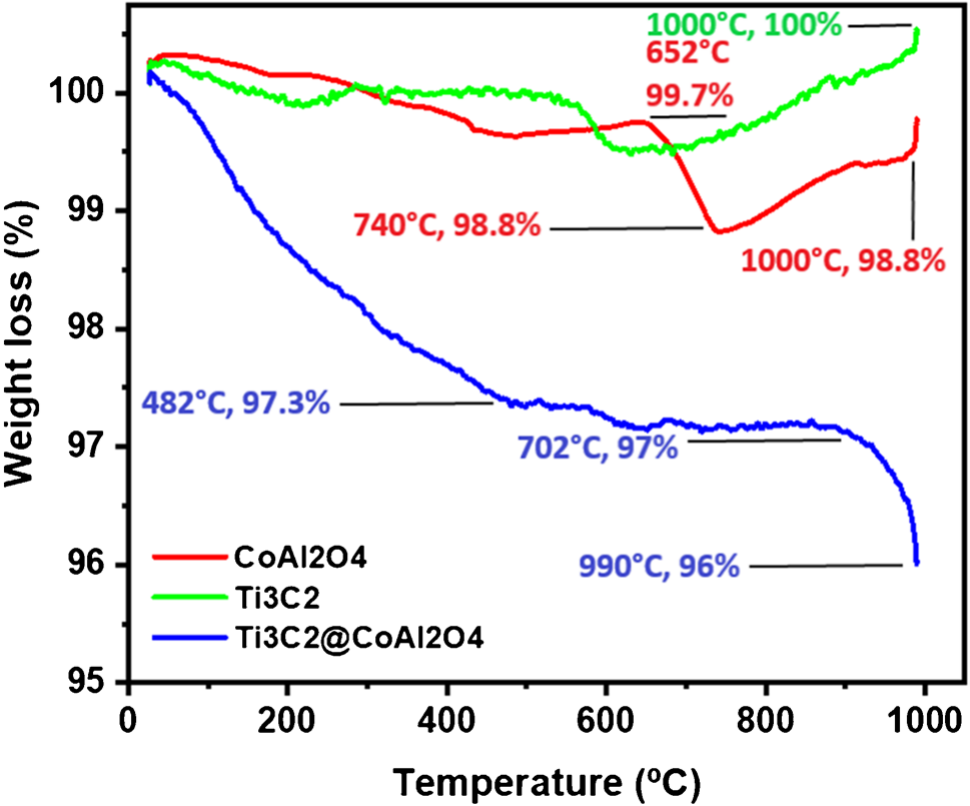
TGA of as-prepared CoAl_2_O_4_, Ti_3_C_2_ MXene, and Ti_3_C_2_@CoAl_2_O_4_ nanocomposite

The TGA of CoAl_2_O_4_, Ti_3_C_2_, and Ti_3_C_2_@ CoAl_2_O_4_ nanocomposites is shown in Fig. 5. Up to 1000 °C, all nanomaterials show excellent thermal stability. CoAl_2_O_4_ does not undergo any weight loss when heated to 1000 °C, which may be related to the absence of surface dehydration and its better heat resistance [38]. Ti_3_C_2_ maintains its stability at temperatures as high as 652 °C. It loses approximately 1.2% of its weight at 1000 °C. This is probably the result of oxygen absorption, which leads to the oxidation of titanium to titanium dioxide (TiO_2_) [39]. For Ti_3_C_2_@ CoAl_2_O_4_, weight loss starts at 482 °C, gradually reaching 97.3% of its original weight by 702 °C. Four percent of the weight was evaporated at 990 °C. Ti_3_C_2_ presents oxidation behavior at high temperatures, while CoAl_2_O_4_ maintains its status as the most thermally stable material. Although the nanocomposite matches the characteristics of its initial components, it is less thermally stable than pure CoAl_2_O_4_.

### *Electrochemical characterization of Ti*_*3*_*C*_*2*_*@CoAl*_*2*_*O*_*4*_*/GCE*

**Fig. 6:**
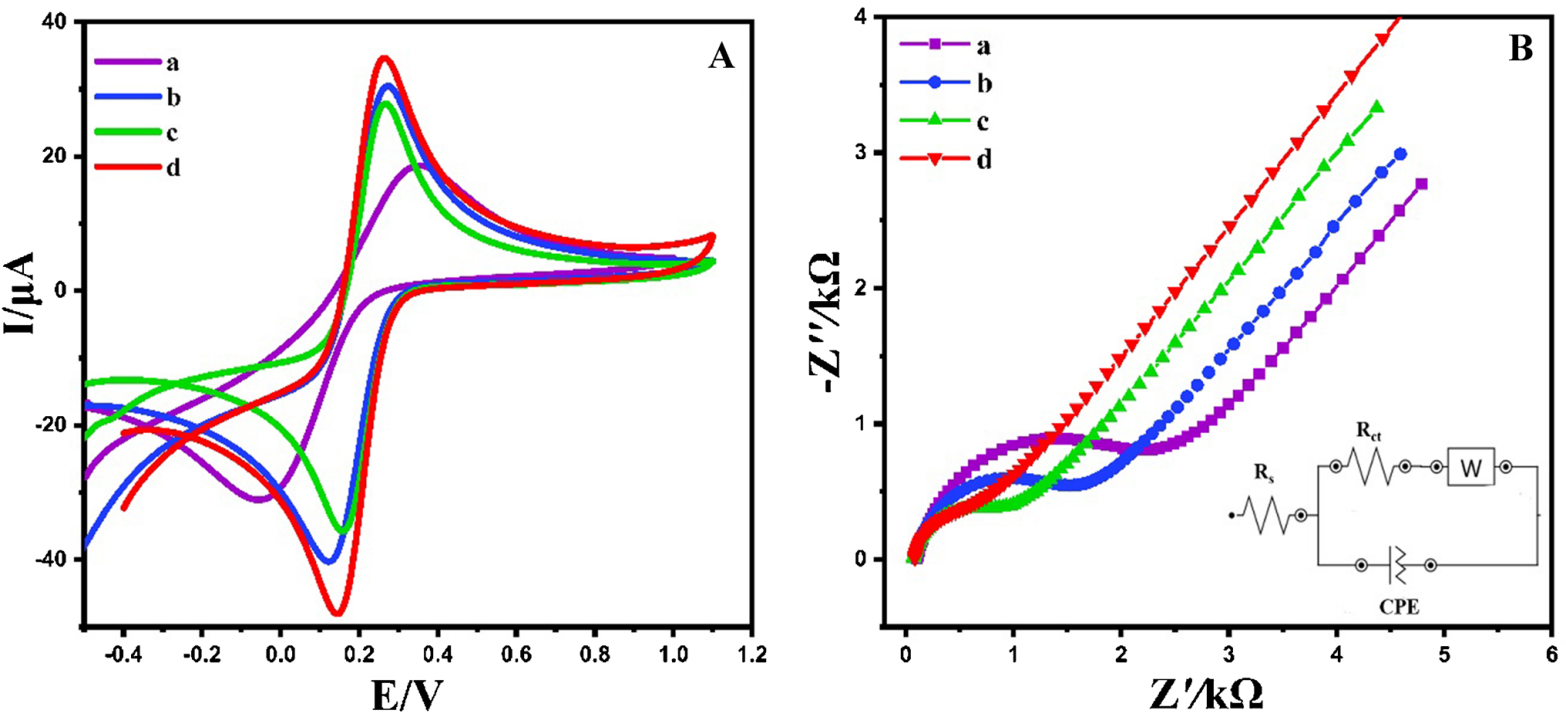
(**A**) CV and (**B**) EIS spectra of the unmodified GCE (a), CoAl_2_O_4_/GCE (b), Ti_3_C_2_/GCE (c), and Ti_3_C_2_@CoAl_2_O_4_/GCE (d); the inset is the equivalent circuit for the EIS test of Ti_3_C_2_@CoAl_2_O_4_/GCE (Rs, electrolyte solution resistance; Rct, element of interfacial electron transfer resistance; CPE, constant phase element; W, Warburg impedance resulting from the diffusion of ions)

The CV and EIS methods were employed to evaluate the surface properties of different electrodes (Fig. 6). A solution of [Fe (CN)₆]^3^⁻^/4^⁻ (5.0 mM) in 0.1 M potassium chloride served as the electrolyte for this analysis. Figure 6A illustrates the CV profiles of the unmodified GCE, CoAl_2_O_4_/GCE, Ti_3_C_2_/GCE, and Ti_3_C_2_@CoAl_2_O_4_/GCE electrodes (50 mV s⁻^1^). The peak separations (ΔEp) were determined as 0.38, 0.13, 0.11, and 0.11 V for the unmodified GCE, CoAl_2_O_4_/GCE, Ti_3_C_2_/GCE, and Ti_3_C_2_@CoAl_2_O_4_/GCE, respectively. Furthermore, the Ti_3_C_2_@CoAl2O4/GCE exhibited a significant enhancement in reduction peak currents along with a shift toward lower reduction potential, highlighting the superior electrocatalytic activity of the synthesized composite material.

As illustrated in Fig. S1A–B, the electroactive surface area (EASA) of unmodified GCE, CoAl_2_O_4_/GCE, Ti_3_C_2_/GCE, and Ti_3_C_2_@CoAl_2_O_4_/GCE was determined using the Randles–Ševćik equation (Eq. 1), which establishes a correlation between the square root of the scan rate (*v*^1^/^2^) and the anodic peak current (*I*_pa_) in a 5 mM [Fe(CN)₆]^3^⁻/^4^⁻ solution [40].

1 $$I=\left(2.69\times {10}^{5}\right){ n}^\frac{3}{2} A {D}^\frac{1}{2} {v}^\frac{1}{2} {C}_{0}$$

*A* represents the electrode area (cm^2^), *D* shows the diffusion coefficient (cm^2^ s^−1^), *n* is the number of electrodes (*n* = 1), *v* exhibits the potential scan rate (V s^−1^), and *C*_0_ is the concentration (mol cm^−3^).

This electrochemical system was selected due to its well-known reversible redox behavior, which allows for accurate assessment of the electrode surface area. The EASA was calculated as 0.076 cm^2^ for the unmodified GCE, 0.099 cm^2^ for CoAl_2_O_4_/GCE, 0.12 cm^2^ for Ti_3_C_2_/GCE, and 0.15 cm^2^ for Ti_3_C_2_@CoAl_2_O_4_GCE, indicating a substantial enhancement in surface activity with the composite modification. The enhanced surface area of the Ti_3_C_2_@CoAl_2_O_4_/GCE is attributed to the incorporation of Ti_3_C_2_ and CoAl_2_O_4_, which contribute to a greater number of electroactive sites, thereby improving the electrode's overall electrochemical performance. This larger surface area facilitates better interaction with analytes, leading to a higher current response, which is crucial for enhancing the sensitivity and efficiency of the sensor.

EIS was used to investigate the interfacial properties of different electrodes with a potential of 0.1 V and a frequency range of 10 kHz to 0.1 Hz. The Nyquist plots for the unmodified GCE (a) and Ti_3_C_2_@CoAl_2_O_4_-modified GCE (b) in a 5-mM solution of [Fe (CN)₆]^3⁻/4⁻^ in 0.1 M potassium chloride (KCl) are presented in Fig. 6B.

A simplified Randles equivalent circuit was employed to characterize the electrified interface, incorporating key parameters such as solution resistance (Rs), charge transfer resistance (Rct), and Warburg impedance (Zw), as depicted in Fig. 6B (inset). In this model, impedance variations at different frequencies are represented through a Nyquist plot, where the real component is plotted along the *X*-axis and the imaginary component along the *Y*-axis. The resistance of the interface is reflected in the high-frequency real-axis value, while variations in the slope magnitude become evident at lower frequencies [41].

The charge transfer resistance (Rct), which represents the resistance associated with charge transfer processes at the electrode interface, governs the electron transfer kinetics. The semicircle diameter in the Nyquist plot indicates the Rct value, with larger semicircles corresponding to slower electron transfer kinetics. Rct is influenced by the dielectric and insulating properties of the electrode/electrolyte interface, which vary based on surface modifications. The Rct values were determined as 2900 Ω for the unmodified GCE, 1800 Ω for CoAl_2_O_4_/GCE, 1160 Ω for Ti_3_C_2_/GCE, and 890 Ω for Ti_3_C_2_@CoAl_2_O_4_/GCE. The significantly lower Rct of the Ti_3_C_2_@CoAl_2_O_4_/GCE indicates enhanced conductivity and improved electron transfer efficiency, demonstrating the superior electrochemical performance of the composite material.

The electrocatalytic activity of the designed sensor was evaluated by calculating the standard exchange current density (*I*₀) from Rct using Eq. (S1). The *I*₀ values were determined as 8.83 × 10⁻⁶ µA cm⁻^2^ for the unmodified GCE, 14 µA cm⁻^2^ for CoAl_2_O_4_/GCE, 22.1 µA cm⁻^2^ for Ti_3_C_2_/GCE, and 28.8 µA cm⁻^2^ for Ti_3_C_2_@CoAl_2_O_4_/GCE. This significant enhancement is attributed to the composite electrode’s larger electroactive surface area and the presence of additional functional groups, which facilitate electron transfer and improve electrocatalytic performance.

### Optimization of supporting electrolytes and pH

Buffers used as supporting electrolytes are crucial for enhancing the electro-oxidation of analytes at the modified GCE surface, making the selection of an optimal buffer essential for optimizing experimental conditions. To identify the most effective buffer for CAR detection, DPV measurements were performed using 0.07 mM CAR with a variety of buffers, including Britton–Robinson (BR), phosphate-buffered saline, potassium chloride (KCl), hydrochloric acid (HCl), and sodium hydroxide (Fig. S2C). Among these, BR exhibited the highest anodic peak current (*I*_pa_), demonstrating its superior performance as a supporting electrolyte. Therefore, 0.1 M BR was chosen as the optimal buffer for all subsequent experiments.

**Fig. 7:**
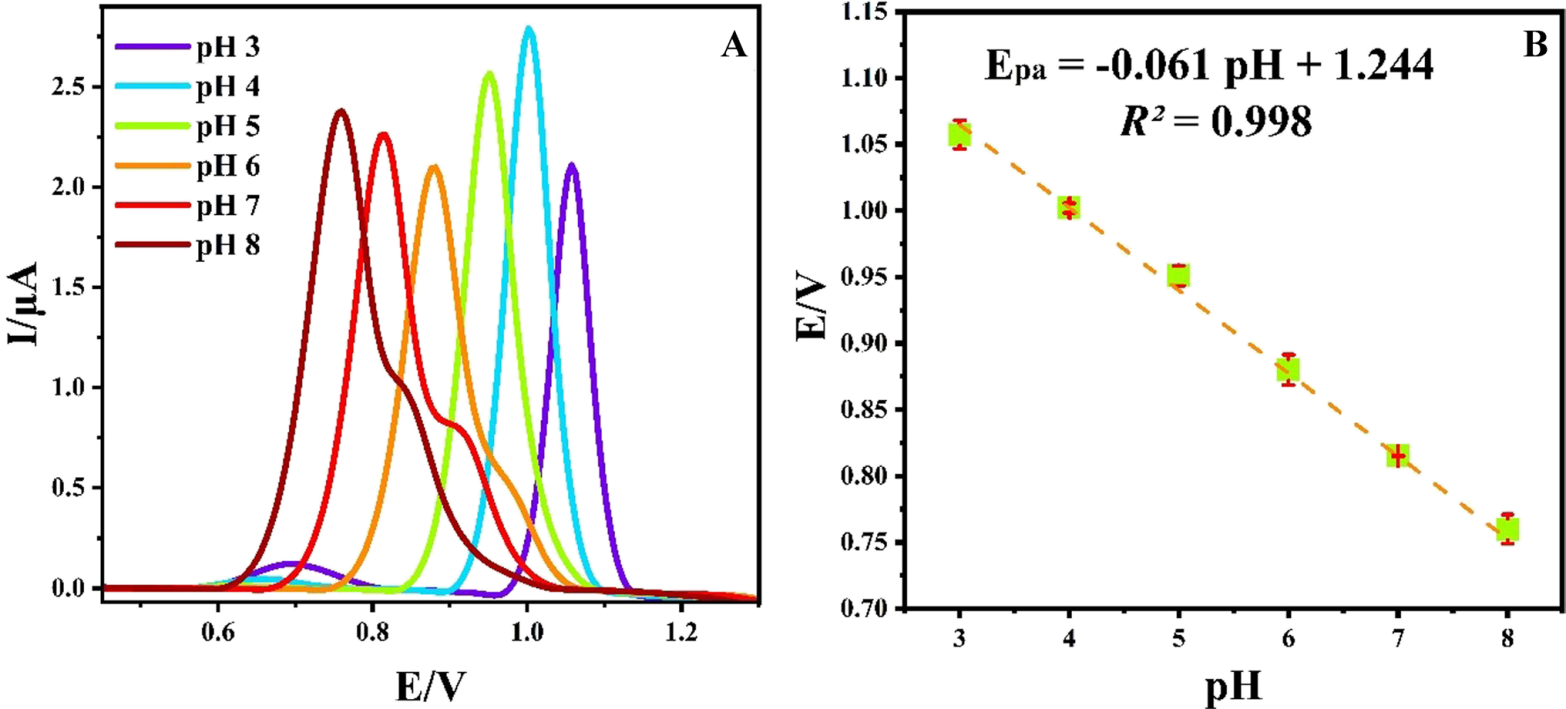
(**A**) DPV voltammograms of 0.07 mM CAR at various pH (3.0–8.0) of BR on the Ti_3_C_2_@CoAl_2_O_4_/GCE and (**B**) the impact of pH on the peak potential (*E*_pa_) of CAR

The pH of the supporting electrolyte significantly influences the reaction mechanism, peak potential, and peak current of the target analyte. DPV experiments were performed at varying pH levels (3.0–8.0) of BR to elucidate the oxidation mechanism of CAR at the Ti_3_C_2_@CoAl_2_O_4_/GCE surface. As illustrated in Fig. 7A, the maximum analytical response was observed at pH 4, which was subsequently chosen as the optimal pH for further experiments. The maximum electrochemical response was observed in BR at pH 4, which can be attributed to the protonated form of cariprazine at this pH, enhancing its interaction with the electrode surface and facilitating electron transfer.

A linear relationship between pH and oxidation potential (Fig. 7B) was noted, with the potential shifting negatively as the pH increased. Using the Nernst equation, the number of protons involved in the electrochemical oxidation process was calculated, revealing an electron-to-proton ratio of 1. This finding indicates an equal participation of electrons and protons in the oxidation of CAR at the modified electrode surface.

### *Study of the Ti*_*3*_*C*_*2*_*@CoAl*_*2*_*O*_*4*_* modifier amount and concentration*

The concentration and amount of Ti_3_C_2_@CoAl_2_O_4_ applied to the electrode surface play a crucial role in modulating the electrochemical response of CAR. To investigate this, the effect of varying concentrations of Ti_3_C_2_@CoAl_2_O_4_ (0.5–2.0 mg mL^−1^) on the electrochemical behavior of 0.07 mM CAR was systematically studied (Fig. S2A). The results indicated that the electrochemical response of CAR improved with increasing Ti_3_C_2_@CoAl_2_O_4_ concentration, reaching an optimal performance at 1.0 mg mL^−1^. In contrast, at higher concentrations (more than 1.0 mg mL^−1^), a noticeable reduction may be attributed to the accumulation or overgrowth of accessible binding sites. Thus, 1.0 mg mL^−1^ was identified as the optimal concentration for further studies.

In addition to optimizing the concentration of the Ti_3_C_2_@CoAl_2_O_4_ composite, the amount of this composite applied to the electrode surface was systematically investigated. For this purpose, different volumes (ranging from 2.0 to 6.0 μL) of a 1.0 mg mL^−1^ Ti_3_C_2_@CoAl_2_O_4_ solution were drop-cast onto the GCE to assess the effect on the voltammetric detection of 0.07 mM CAR at pH 4.0 (Fig. S2B). The electrochemical response was evaluated using DPV, and the resulting peak currents (*I*_pa_) were recorded. The optimization studies revealed that a volume of 2.0 μL produced the highest anodic peak current, suggesting the most favorable electrode modification. This result can be attributed to the optimal amount of the composite material that facilitated efficient electron transfer at the electrode surface. However, increasing the volume beyond 2.0 μL led to a noticeable decrease in the current response. This decline is likely due to the excessive loading of the composite material, which could cause diffusion limitations and hinder the efficient transport of the analyte to the electrode surface. Therefore, 2.0 μL of Ti_3_C_2_@CoAl_2_O_4_ solution was selected as the optimal volume for electrode modification, ensuring both a high electrochemical response and efficient electroactive surface area utilization in subsequent experiments.

### Effect of scan rate

**Fig. 8:**
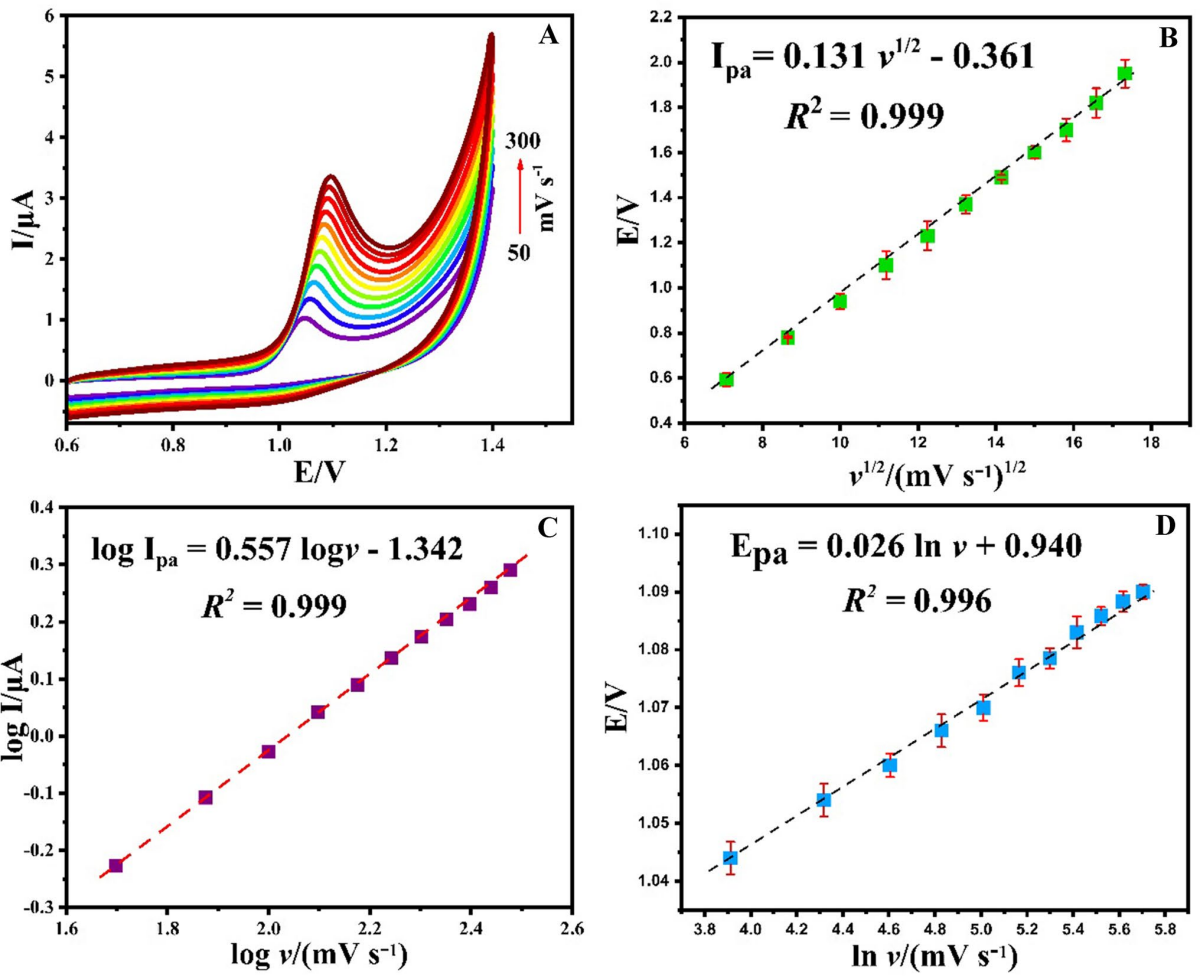
(**A**) Scan rate effect of 0.07 mM CAR, (**B**) plot of *I*_pa_ (μA) vs. *v*^1/2^ (mV s^−1^)^1/2^, (**C**) plot of log *I*_pa_ (μA) vs. log *v* (mV s^−1^), and (**D**) plot of *E*_pa_ vs. ln *v*

The influence of scan rate on the peak current (*I*_pa_) and peak potential (*E*_pa_) at the Ti_3_C_2_@CoAl_2_O_4_/GCE was investigated using CV method in 0.1 M BR (pH 4.0) containing 0.07 mM CAR at scan rates ranging from 50 to 300 mV s⁻^1^ (Fig. 8A). A positive shift in *E*_pa_ with increasing scan rates was observed, indicative of an irreversible electrochemical reaction. As depicted in Fig. 8B, the anodic peak current (*I*_pa_) exhibited a linear relationship with the square root of the scan rate (ν^1/2^), described by the equation *I*_pa_ = 0.131 ν^1/2^ − 0.361 (*R*^2^ = 0.999). This confirms that the oxidation process at the Ti_3_C_2_@CoAl_2_O_4_-modified GCE surface is diffusion controlled [42].

Further analysis of log (*I*_pa_) versus log(ν) (Fig. 8C) yielded a linear relationship with a slope close to 0.5, consistent with a diffusion-controlled electrode process [43]. Additionally, the peak potential (*E*_pa_) displayed a linear relationship with the natural logarithm of the scan rate (ln ν), expressed as *E*_pa_ = 0.026 ln ν + 0.940 (*R*^2^ = 0.996) (Fig. 8D). Based on the slope of the *E*_pa_ versus ln ν plot and following Laviron's equation for an irreversible and reaction (Eq. 2), the number of electrons (*n*) involved in the reaction was determined to be 2.

2 $${E}_{\text{pa}}={E}^{^\circ }+\left(\frac{RT}{\alpha nF}\right)\text{ln}\left(\frac{RTK^\circ }{\alpha nF}\right)+\left(\frac{RT}{\alpha nF}\right)\text{ln}v$$

Here, *E*° is the formal redox potential, α is the charge transfer coefficient, *n* is the number of electrons transferred, *K*° is the standard heterogeneous rate constant, and *v* is the scan rate.

**Scheme 1:**
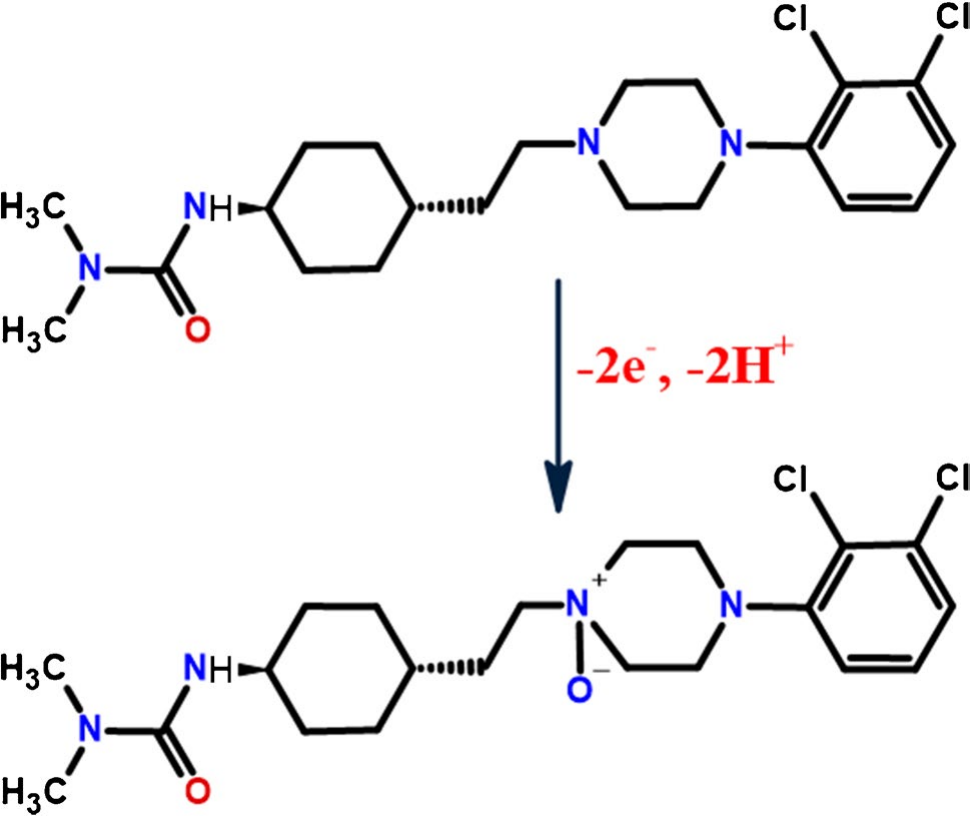
The plausible electrooxidation process of CAR

These results collectively indicate that the oxidation of CAR at the Ti_3_C_2_@CoAl_2_O_4_/GCE surface involves the transfer of two electrons and two protons. The proposed electrooxidation pathways for CAR under these conditions are presented in Scheme 1 [44].

### Analytical performance

#### ***Quantification of CAR at the surface of Ti***_***3***_***C***_***2***_***@CoAl***_***2***_***O***_***4***_***/GCE***

**Fig. 9:**
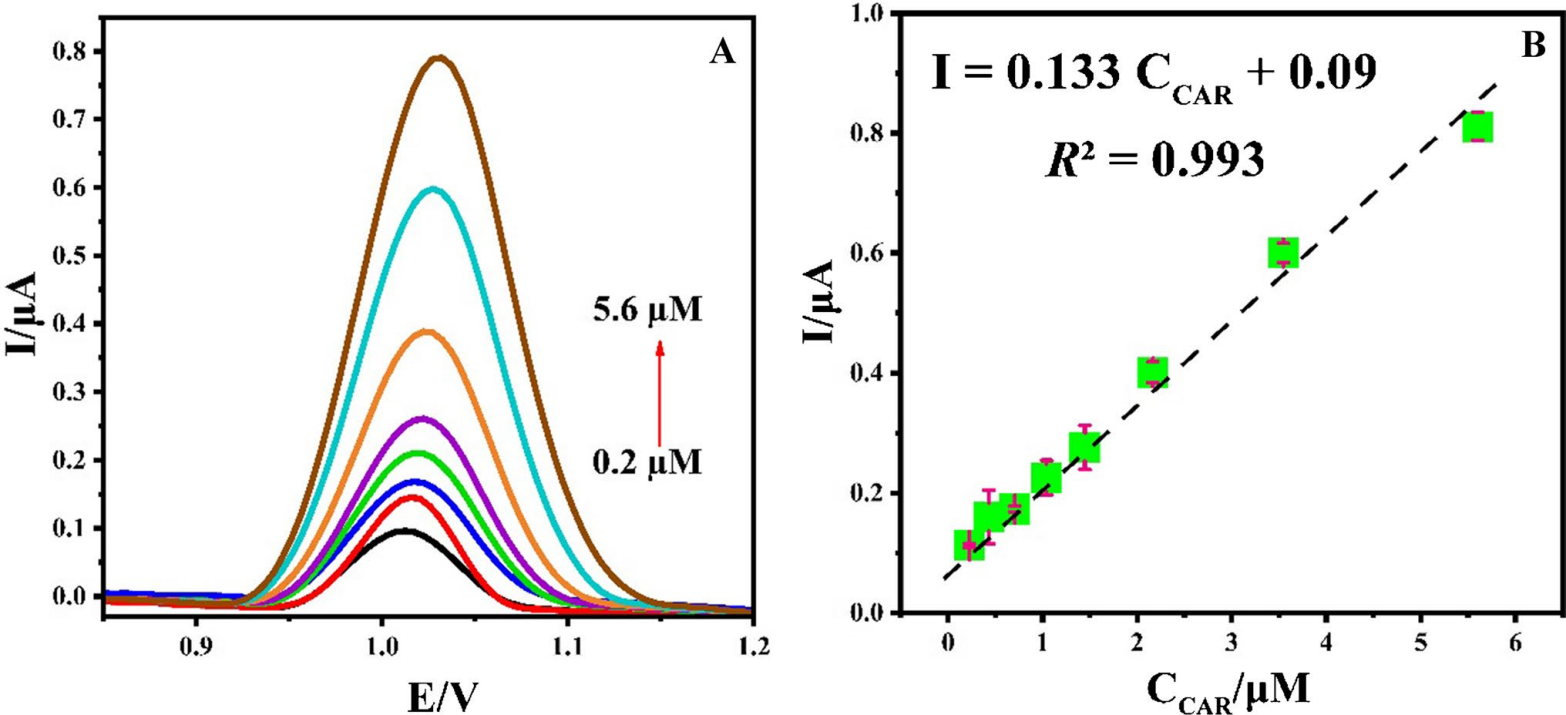
(**A**) DPVs of Ti_3_C_2_@CoAl_2_O_4_/GCE in BR (0.1 mol L^−1^, pH = 4.0) containing various concentrations of CAR

In this study, the Ti_3_C_2_@CoAl_2_O_4_-modified GCE was employed to quantify various concentrations of CAR using DPV under optimal conditions (BR, pH 4.0). The oxidation peak current exhibited a linear relationship with CAR concentration in the range of 0.2–5.6 μM, as illustrated in Fig. 9. The corresponding linear regression equation was determined as *I*_pa_ (μA) = 0.133 *C*_CAR_ (μM) + 0.09 with a correlation coefficient of *R*^2^ = 0.993. The limit of detection (LOD) was calculated as 0.02 µM using the equation LOD = 3σ/*S*, where σ represents the standard deviation of ten blank measurements and *S* is the slope of the calibration curve. Similarly, the limit of quantification (LOQ) was determined to be 0.07 µM based on the equation LOQ = 10σ/*S*. The corresponding characteristics of the linear regression analysis are provided in Table 1. In addition, the sensitivity of the Ti_3_C_2_@CoAl_2_O_4_-modified GCE was calculated to be 0.88 µA µM⁻^1^ cm⁻^2^, further highlighting its excellent performance for the quantification of CAR.

**Table 1:** Analytical performance data for the developed Ti_3_C_2_@CoAl_2_O_4_/GCE

Parameter	Value
Linearity range (µM)	0.2–5.6
Slope (μA/µM)	0.133
Intercept (μA)	0.09
Correlation coefficient (*R*^2^)	0.993
LOD (µM)	0.02
LOQ (µM)	0.07
Standard error of slope	0.005
Standard error of intercept	0.012

These findings highlight the remarkable sensitivity of the Ti_3_C_2_@CoAl_2_O_4_/GCE, demonstrating its potential for the accurate and reliable detection of CAR.

Table S1 presents a comparative analysis of CAR quantification, assessing the proposed sensor alongside the reported methods based on key analytical parameters, including the detection technique, electrode modification, linear dynamic range, LOD, and applicability to real samples.

#### ***The repeatability, reproducibility, and interference study of the Ti***_***3***_***C***_***2***_***@CoAl***_***2***_***O***_***4***_***/GCE***

The performance of the Ti_3_C_2_@CoAl_2_O_4_-modified GCE was validated by evaluating its repeatability and reproducibility using DPV measurements in a 0.1 M BR (pH 4.0) containing 0.7 μM CAR. Repeatability was assessed by performing nine consecutive measurements with the same electrode (Fig. S3A), while reproducibility was examined by comparing DPV responses from ten independently prepared sensors (Fig. S3B). The sensor exhibited excellent stability, with RSD of 2.9% for repeatability and 2.8% for reproducibility, confirming its reliability.

The selectivity of the electrode for CAR detection was also tested by introducing 0.7 μM CAR into a 0.1 M BR solution (pH 4.0) and recording its DPV curve (Fig. S4). Subsequently, various common substances found in drugs and biological fluids, such as sodium sulfate (Na₂SO₄), potassium chloride, d-glucose, l-arginine, l-methionine, potassium nitrate, uric acid, and caffeine, were added at concentrations 100 times higher than that of CAR. The results showed less than a 3% variation in the peak current of CAR (Fig. S5), demonstrating that the Ti_3_C_2_@CoAl_2_O_4_-modified GCE exhibits excellent selectivity for CAR detection.

#### Determination of CAR in biological and pharmaceutical samples

The performance of the developed Ti_3_C_2_@CoAl_2_O_4_-modified GCE was further evaluated by determining the concentrations of CAR in human blood serum, human urine, and CAR tablets using the DPV method. Recovery values were calculated, and the results are summarized in Table 2. The recovery percentages ranged from 98.52 to 103.94%, with RSD of less than 3%. These findings confirm the reliability and accuracy of the proposed sensor for the analytical quantification of CAR in real samples, demonstrating its potential for applications in clinical and pharmaceutical settings.

**Table 2:** Measurement of CAR in human blood serum, human urine, and tablets

Sample	Added (μM)	Found (μM)^a^	RSD (%)	Recovery (%)
Human blood serum	0.8 0.9 1	0,83 0,93 1.01	1.0 1.7 1.5	103.8 103.94 101.42
Human urine	0.8 0.9 1	0.79 0.91 1	0.7 0.4 1.8	99.02 101.85 100.55
Tablet	0.8 0.9 1	0.82 0.88 1.02	3.6 2.9 2.7	103.4 98.52 102.9

^a^Average of three replicate measurements

## Conclusion

This study successfully developed a novel electrochemical sensor for the detection of CAR by modifying a GCE with a Ti_3_C_2_@CoAl_2_O_4_ nanocomposite. The integration of MXene (Ti_3_C_2_) and spinel nanostructure (CoAl_2_O_4_) materials synergistically improved the sensor's conductivity, surface area, and electroactive sites, resulting in superior sensitivity, selectivity, and efficiency. The sensor exhibited excellent stability, reproducibility, and selectivity while demonstrating high sensitivity in the detection and quantification of CAR across pharmaceutical and biological samples. The successful application to these samples further underscores its practical relevance and potential for widespread use. Offering cost-effectiveness and superior performance, this innovative sensor represents a significant advancement in analytical methods, providing a promising tool for clinical and research applications in CAR detection.

## Supplementary Information

Below is the link to the electronic supplementary material.

ESM 1 (DOCX 866 KB)

## Data Availability

No datasets were generated or analysed during the current study.
