# Supplementary material for: Highly sensitive electrochemical determination of cariprazine using a novel Ti3C2@CoAl2O4 nanocomposite: application to pharmaceutical and biological sample analysis
Source: Mikrochim Acta. 2025 Mar 24;192(4):248. doi: 10.1007/s00604-025-07104-1 (PMC11933176; doi:10.1007/s00604-025-07104-1)
Supplement: Supplementary file 1 — (DOCX 866 KB) [file 604_2025_7104_MOESM1_ESM.docx]

**Supplementary Information**

**Highly Sensitive Electrochemical Determination of Cariprazine Using a Novel Ti_3_C_2_@CoAl_2_O_4_** **Nanocomposite: Application to Pharmaceutical and Biological Sample Analysis**

***Elif Naz Öven ^a b*^, Asena Ayse Genc ^a b^, Nevin Erk ^a*^, Wiem Bouali ^a b^, Qamar Salamat ^c^, and Mustafa Soylak ^c d e^***

^a^ Ankara University, Faculty of Pharmacy, Department of Analytical Chemistry, 06560 Ankara, Turkey

^b^ Ankara University, The Graduate School of the Health Sciences, 06110 Ankara, Turkey

^c^ Erciyes University, Faculty of Sciences, Department of Chemistry, 38039, Kayseri, Turkey

^d^ Technology Research & Application Center (TAUM), Erciyes University, 38039, Kayseri, Turkey

^e^ Turkish Academy of Sciences (TUBA), Cankaya, Ankara, Turkey

Email corresponding author: [erk@pharmacy.ankara.edu.tr](mailto:erk@pharmacy.ankara.edu.tr)

elifnazoven0@gmail.com

**Materials and Reagents**

Deionized water (DI) (Millipore, USA) with a conductivity of 18.2 MΩ cm was used as a source of water. Pristine nanodiamond, aluminum nitrate nonahydrate (Al(NO_3_)_3_.9H_2_O), copper (II) nitrate trihydrate (Cu(NO_3_)_2_.3H_2_O), citric acid, iron (II) sulfate heptahydrate (FeSO_4_.7H_2_O), iron chloride hexahydrate (FeCl_3_.6H_2_O), ammonia solution, nitric acid, and sulphuric acid were obtained from Merck company (Darmstadt, Germany).

Glucose (99.5 %), L-arginine (98.0 %), L-methionine, sodium hydroxide, potassium hexacyanoferrate (K_3_Fe(CN)_6_, 99.5 %), hydrochloric acid, sodium Acetate, ascorbic acid, uric acid (99.0 %), acetic acid, potassium chloride, sodium phosphate, sodium sulfate, potassium chloride, sodium sulfate, were purchased from Sigma Aldrich Co. (https://www.sigmaaldrich.com, Germany). Britton-Robinson buffer was made of boric acid, phosphoric acid, potassium chloride, and acetic acid solutions. Pure Cariprazine powder was purchased from Sigma-Aldrich (98%). The stock solution of CAR was prepared in deionized water and methanol (1:1). Human plasma samples were also acquired from Sera-Flex Inc. All chemical compounds were analytical grade and used without additional refinement.

**Apparatus**

Voltammetric experiments were carried out using AUTO LAB system with PGSTAT204 electrochemical workstation (Metrohm Inc., Switzerland) with a glassy carbon electrode system in a one-compartment of 10 mL electrochemical cell. All electrochemical measurements were performed at 25 ^◦^C unless otherwise specified. The pH of the supporting electrolyte was monitored with a pH meter (Hanna Instruments, Woonsocket, Rhode Island, USA).

The FE-SEM and EDX characterizations were perused by the Zeiss Gemini 500 FE-SEM (Germany). The XRD (Bruker AXS D8 Advance, Germany) analyses were performed with monochromatic Cusingle bondK radiation (= 1.541) at 40 kV utilizing a diffractometer together. The adsorbents were also perused by Thermo Nicolet 5700 Fourier transform infrared (FTIR) spectroscopy.


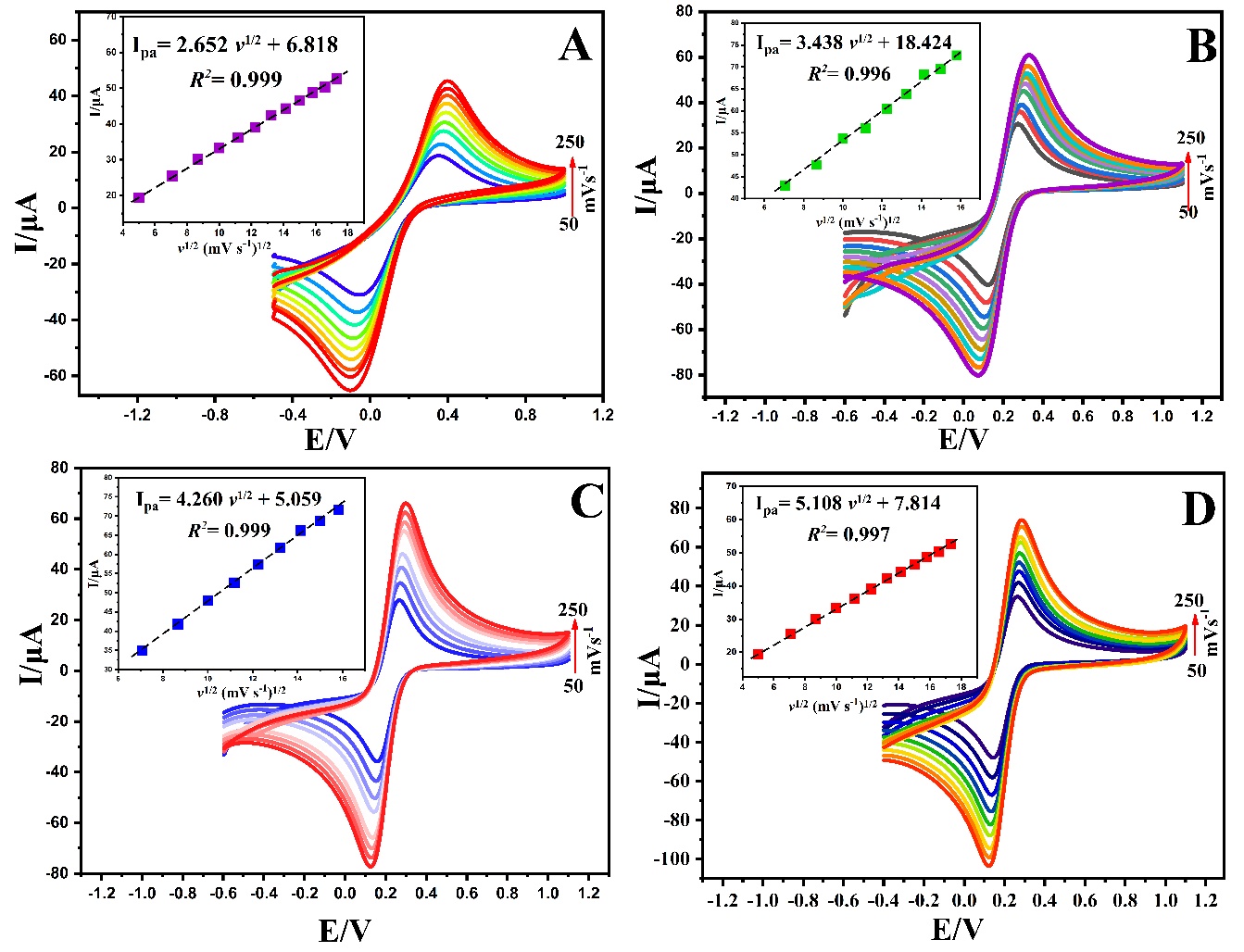


**Figure S1.** The recorded CV curves at various scan rates in the presence of 5.0 mM [Fe (CN) _6_]^3−/4−^ containing 0.1 M KCl on Bare/GCE (A), CoAl_2_O_4_/GCE (B), Ti_3_C_2_/GCE (C), and Ti_3_C_2_@CoAl_2_O_4_/GCE (D).

$$I_{0}= \frac{RT}{n F R_{ct}} (S1)$$

I_0_ is the standard exchange current density (A cm^-2^), R stands for the universal gas constant (8.314 J K^–1^ mol^–1^), T is the temperature (298.15 K), F is the Faraday constant (96485 C mol^–1^), R_ct_ is the electron transfer resistance (Ω), and n is the number of electrons transferred. .


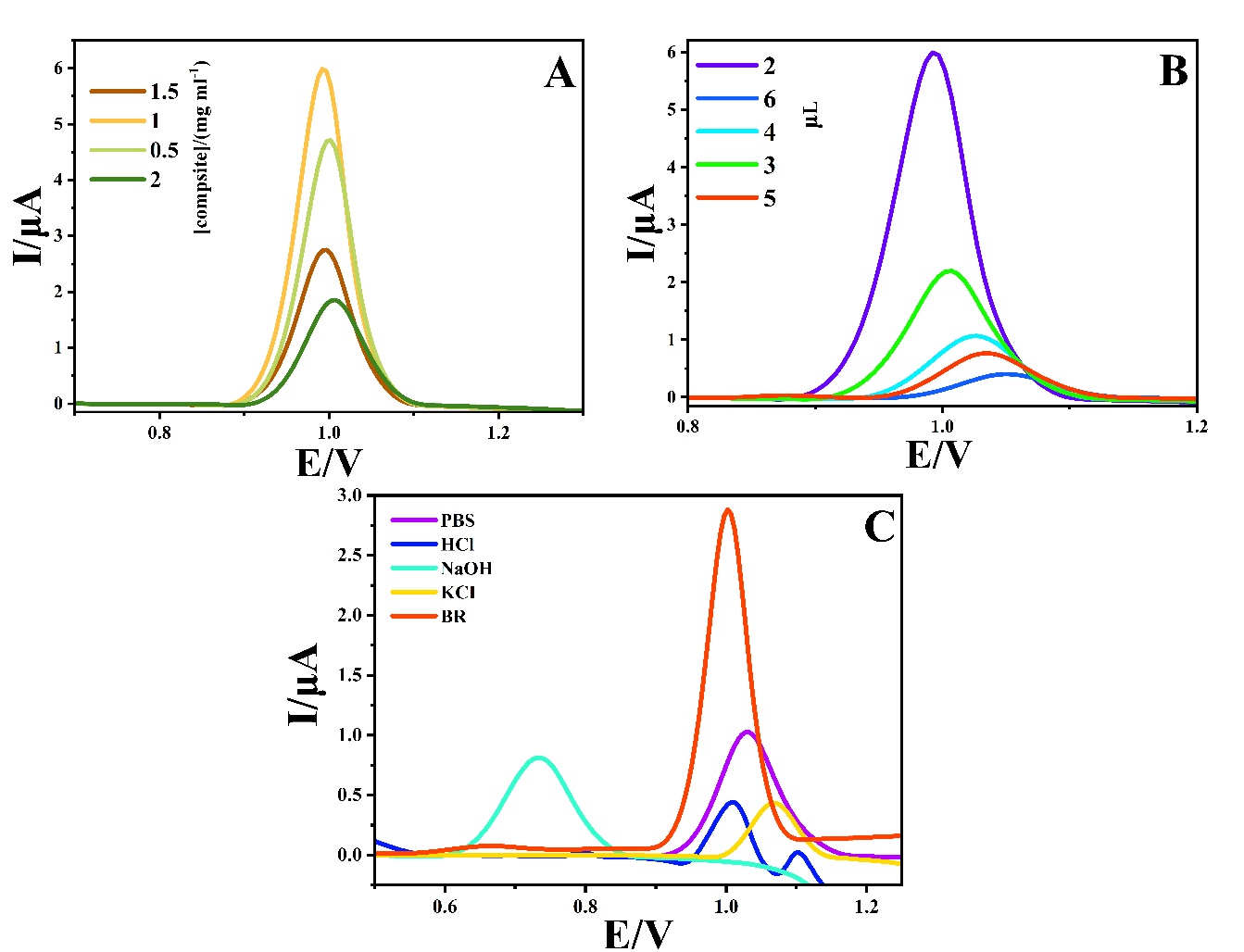


**Fig. S2.** Influence of concentration of composite (A), amount of composite (B), and supporting electrolyte (C), on the oxidation peak currents of 0.07 mM CAR.

**Table S1.** Comparison between the proposed method for determination of CAR to previously reported methods.

| **Method** | **Modifier** | **Linear Range** | **LOD** | **Application** | **Ref** |
| --- | --- | --- | --- | --- | --- |
| DPV | Bare/GCE | 2.5 µM–50 µM | 0.64 µM | Human serum and tablets. | [1] |
| DPV | MIP/GCE | 0.1–2.5 pM | 12.7 fM | Human serum and tablets. | [1] |
| DPV | COF-PTA@CuCoFe-LDH/GCE | 0.2–5.6 µM | 0.02 µM | Human serum, human urine and tablets. | Our work |


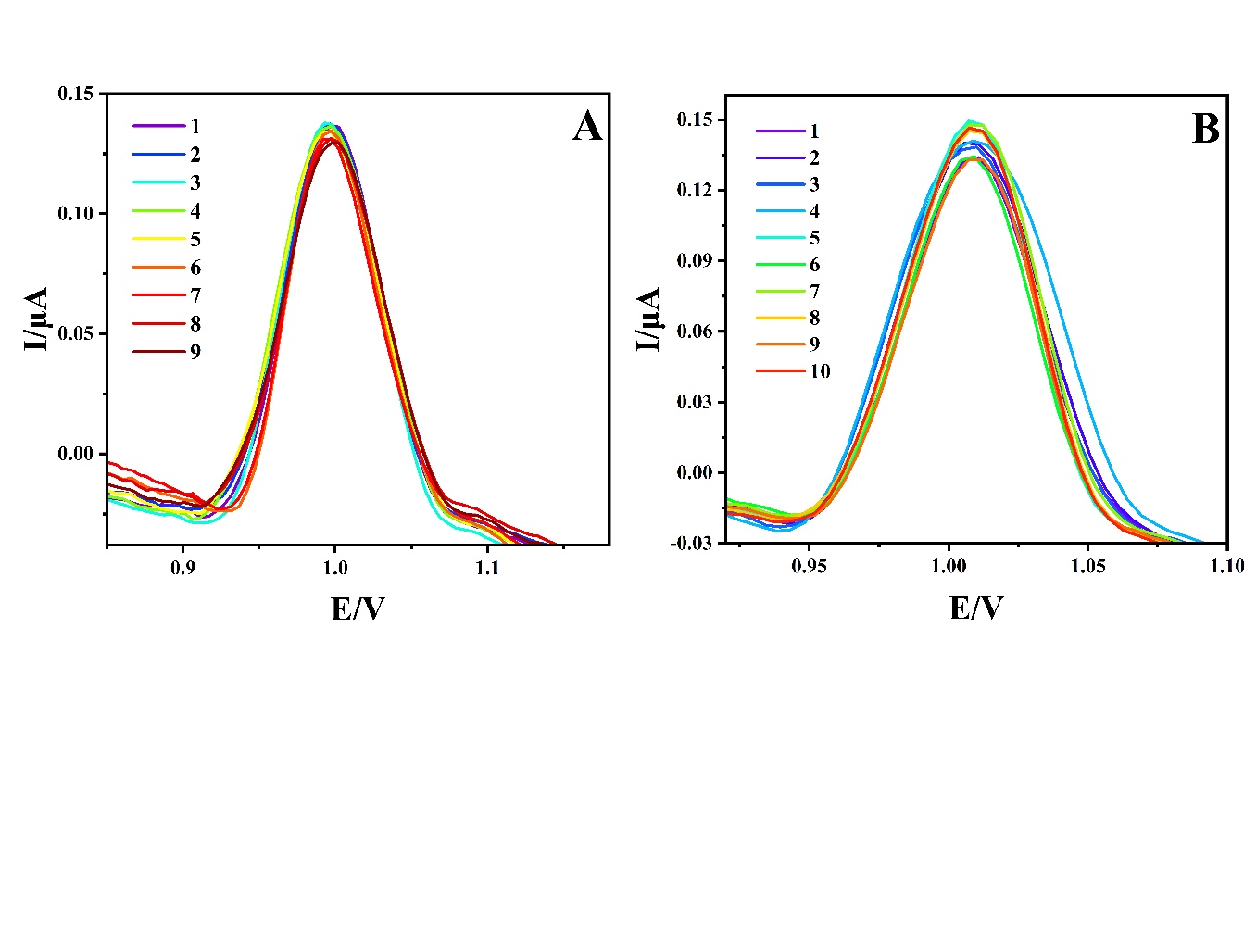


**Fig S3.** Repeatability (A), and reproducibility (B), of 0.7 μM CAR at Ti_3_C_2_@CoAl_2_O_4_ /GCE in B-R buffer (pH 4.0).

**
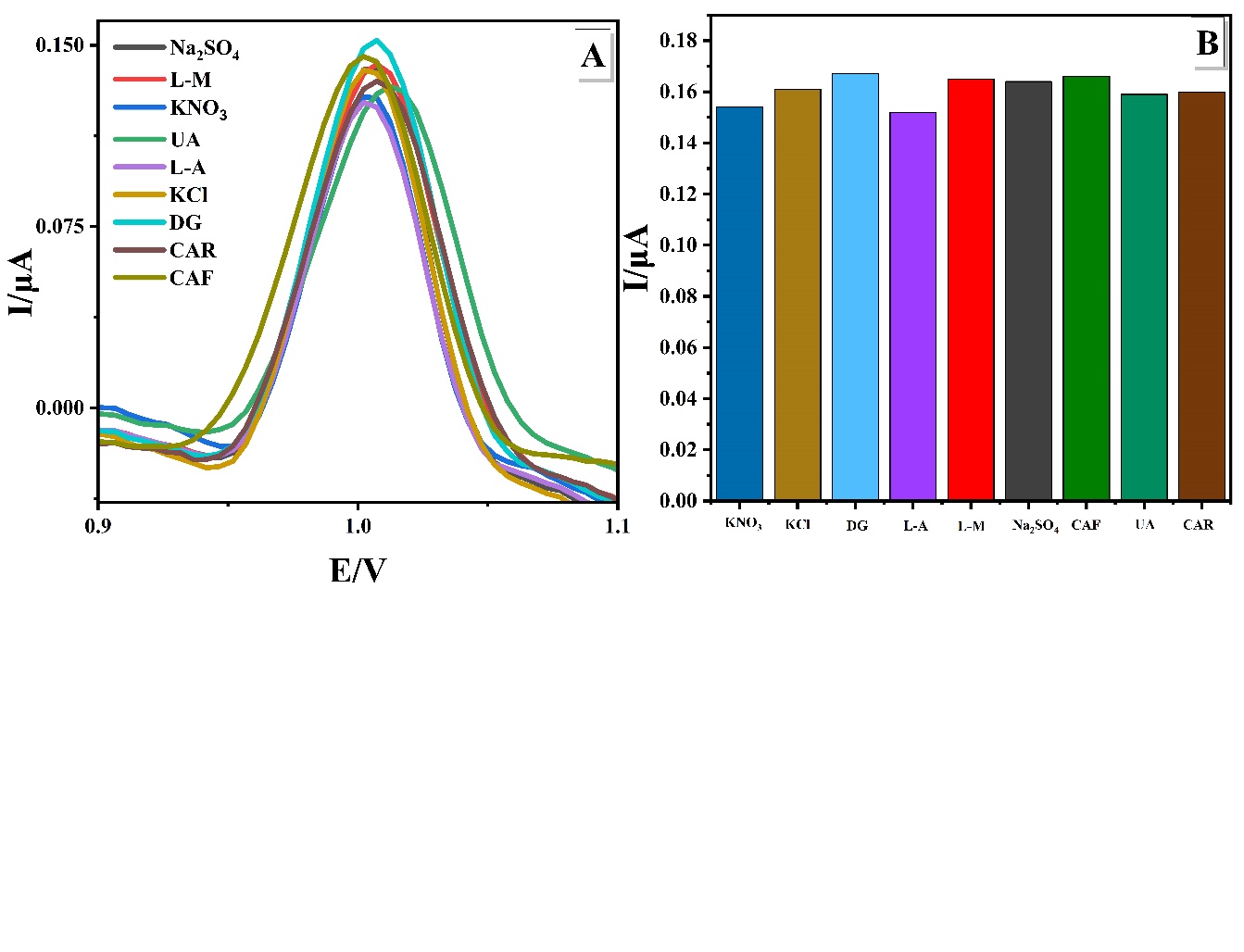
**

**Fig S4.** Selectivity of of 0.7 μM CAR at Ti_3_C_2_@CoAl_2_O_4_ /GCE in B-R buffer (pH 4.0).

**References**

1. Hosseinzadeh, B., Cetinkaya, A., Atici, E. B., & Ozkan, S. A. (2024). Detection of the antipsychotic drug cariprazine using a low-cost MIP-based electrochemical sensor and its electrooxidation behaviour. *Microchemical Journal, 207*, 112105.
